# Differential effects of social feedback valence and self-relevance on brain responses and behaviour

**DOI:** 10.1093/scan/nsaf062

**Published:** 2025-06-20

**Authors:** Hanne Helming, Antje Peters, Su Arkun, Maximilian Bruchmann, Robert Moeck, Thomas Straube, Sebastian Schindler

**Affiliations:** Institute of Medical Psychology and Systems Neuroscience, University of Münster, Münster, 48149, Germany; Institute of Medical Psychology and Systems Neuroscience, University of Münster, Münster, 48149, Germany; Otto Creutzfeldt Center for Cognitive and Behavioral Neuroscience, University of Münster, Münster, 48149, Germany; Institute of Medical Psychology and Systems Neuroscience, University of Münster, Münster, 48149, Germany; Institute of Medical Psychology and Systems Neuroscience, University of Münster, Münster, 48149, Germany; Otto Creutzfeldt Center for Cognitive and Behavioral Neuroscience, University of Münster, Münster, 48149, Germany; Institute of Medical Psychology and Systems Neuroscience, University of Münster, Münster, 48149, Germany; Institute of Medical Psychology and Systems Neuroscience, University of Münster, Münster, 48149, Germany; Otto Creutzfeldt Center for Cognitive and Behavioral Neuroscience, University of Münster, Münster, 48149, Germany; Institute of Medical Psychology and Systems Neuroscience, University of Münster, Münster, 48149, Germany; Otto Creutzfeldt Center for Cognitive and Behavioral Neuroscience, University of Münster, Münster, 48149, Germany

**Keywords:** EEG/ERPs, social evaluative feedback, learning, expectation, feedback incongruence

## Abstract

Theoretical accounts propose that people update their feedback expectations asymmetrically, with stronger updating after positive than negative feedback and self-relevant than irrelevant feedback. Further, attributions to the senders influence neuronal responses towards social evaluative feedback. In this study, we examined how both attributed self-relevance and acquired sender valence through their feedback behaviour impact learning about and Event-Related Potential (ERP) responses towards the social evaluative feedback. We investigated these questions in an ERP study (*N *= 40), where participants received either constant positive or negative feedback from senders, either self-relevant or directed to an unknown person. Participants first indicated their feedback expectations and were then exposed to the feedback and the sender’s face. Feedback expectations changed according to sender behaviour over time, while surprisingly, expectations changed stronger for negative senders in general and positive self-irrelevant senders. For feedback, increased P1 responses to worse-than-expected feedback were observed, while mid-latency Early Posterior Negativity; Feedback Related Negativity (FRN) and late components Late Positive Potential to feedback were increased by feedback self-relevance. The FRN was additionally affected by sender valence and expectedness. Our findings thereby reveal different facets of behavioural and neuronal effects of attributed sender self-relevance and acquired sender valence.

## Introduction

Social interaction is one primary human motivation and fundamental for developing the self (Andersen et al. 1997). The self is proposed to be based on the integration of evaluations made by significant others (Mead 1934, Lundgren 2004), which is influenced by several sender and receiver characteristics (Collins and Stukas 2006; see also Falk and Scholz 2018). Positive feedback is something most individuals are excited about, and regarding acceptance, positive social evaluative feedback appears vital to fulfilling the psychological need to belong (Baumeister and Leary 1995). Furthermore, positive feedback, and especially unexpected positive feedback, has been shown to elicit updating towards more positive expectations rather asymmetrically and more positively for self-relevant expectations (Hepper et al. 2011). We constantly update our expectations of the feedback based on previous experience, and, at the same time, we shift our attitude towards the sender. For example, confronting participants with the likelihood of different risks leads to a selective updating towards unexpected positive information (e.g. see Sharot et al. 2011, Garrett et al. 2014). Furthermore, participants show stronger updating towards positive social evaluative self­relevant than other-related information (Korn et al. 2012).

A general model for predictive processing in healthy versus depressive individuals by Kube et al. (2020) proposes that when healthy people have negative expectations but receive information that is not affirmative to their negative expectations and positively valenced, they tend to generalize the positive information and update their expectations towards more positive expectations. In contrast, after disconfirmation and negative valenced information, positive expectations elicit cognitive immunization and the longer maintenance of the original expectation (Kube et al. 2020; see also Pinquart et al. 2021). The proposed mechanisms in healthy participants are based on studies contrasting expectations and feedback on performance measures (Kube et al. 2019), awaiting confirmation in social evaluative settings. Here, people are reasoned to update what to be expected from others, enabling them to predict how others like or dislike them, approaching or avoiding certain groups or situations (Schoch et al. 2015, Nikitin and Schoch 2021). Studies show that positive social evaluative feedback enhances the interest in the person providing the feedback (Vanderhasselt et al. 2015) and the likelihood of reciprocal positive behaviour (Kroczek and Mühlberger 2022) or feedback (Weinberg et al. 2021). Negative feedback induces negative assumptions about the sender (Rösler et al. 2023), reduces rated attractiveness (Xie et al. 2022), and increases reciprocal negative behaviour (Kroczek et al. 2021) or negative feedback (Yoon et al. 2018). In previous social evaluative experimental designs, examining learning about specific sender behaviour was impossible given the lack of different behaviour of senders (e.g. see Korn et al. 2012, Elder et al. 2022, Koban et al. 2023) or lack of sender repetitions (e.g. see van der Molen et al. 2017, Will et al. 2017, 2020).

Regarding the concurrent neuronal responses during social evaluative feedback, specific Event-Related Potential (ERPs) were identified as relevant during initial stimulus processing and intermediate and late information processing steps. Early effects, in the P1 and N1 range (∼80–100 ms and ∼120–170 ms post feedback over occipital sensors), are reported in some studies for more self-relevant social evaluative feedback, but in contrast to later ERPs, findings are conflicting and may be based on specific design aspects (e.g. attributed human vs. computer feedback; for review, see Peters et al. 2024). These findings are similar to feedback valence, with no P1 and variable N1 effects (reporting effects Schindler et al. 2021; reporting no effects Schindler and Kissler 2018). The following Early Posterior Negativity (EPN) appears over similar occipital scalp regions, starting around 200 ms post-stimulus, and indexes early top-down or stimulus-driven attention processes for verbal stimuli (e.g. see Schindler and Kissler 2016b). The Feedback-Related Negativity (FRN) occurs between 200 and 300 ms during mid-latency processing, primarily over fronto-central sensors, and is thought to originate in the Anterior Cingulate Cortex (Becker et al. 2014, Hauser et al. 2014). It is more pronounced when feedback is unexpected or unexpectedly negative (Peters et al. 2024). Finally, the Late Positive Potential (LPP) occurs from about 400 ms post-stimulus with elaborate stimulus processing, including stimulus evaluation and explicit emotional appraisal, self-referential processing, and information integration (e.g. see Dolcos and Cabeza 2002, Hajcak et al. 2010). Concerning feedback self-relevance and valence, reliably increased EPN and LPP responses are observed for self-relevant social feedback and emotional compared to neutral social evaluative feedback (e.g. see Rohr and Abdel Rahman 2015, Schindler et al. 2015, Schindler and Kissler 2016a, 2018; see also Peters et al. 2024).

Based on two sessions, this study tested the processing and effects of feedback incongruence with self-view and expectation. In the first session, participants described and rated themselves. In the second session, they were shown six faces of putative senders, giving self-relevant or self-irrelevant social evaluative feedback while electroencephalogram (EEG) was recorded. Based on the self-rating of participants, senders consistently gave either more positive or negative feedback, while the self-irrelevant senders used the feedback values from relevant senders on valence-matching words. We expected participants to update their expectations to a stronger extent when receiving positive feedback, possibly further increased by self-relevance. Concerning ERPs for feedback incongruence, we expected no P1 effects but increased N1, EPN, FRN, and LPP amplitudes for self-relevant evaluative feedback.

## Method

### Participants

A sample of 48 healthy participants was recruited based on similar studies within the field that typically varied between 28 and 50 participants (Schindler and Kissler 2016a, 2018, Schindler et al. 2019a, 2020, 2021). Participants were recruited through the student newsletter (ASTA) and personal networks. Seven participants were excluded due to not completing the second appointment, and one was excluded due to insufficient EEG data quality. The final sample consisted of 40 native-level German speakers (7 males, 32 females, 1 diverse; mean age = 22.80, SD = 2.88, range 18 to 31 years). A one-week time gap between the first and second measurements was planned (*M *= 7.68 days, SD = 1.27; Min = 5, Max = 11). All participants were right-handed, had normal or corrected-to-normal vision, and reported no neurological or psychiatric disorders. All participants provided written informed consent and received €12 per hour of participation or credit points for psychology students. The study was approved by the Deutsche Gesellschaft für Psychologie ethics committee.

### Stimuli

We assigned and matched 180 adjectives to four word lists (see Table 1). Adjectives were pre-rated using the self-assessment manikins (Bradley and Lang 1994) for valence, arousal, concreteness, and self-relevance of personality evaluations. Linguistic properties were matched using the dlex database (Heister et al. 2011). The list assignment to the senders was counterbalanced across participants. Participants filled in different questionnaires, the German versions of the Beck-Depression-Inventory (Beck et al. 2009), the Rosenberg self-esteem scale (von Collani and Herzberg 2003), the Fear of Negative Evaluation Scale (Watson and Friend 1969, Kemper et al. 2011), the Anxiety Sensitivity Scale (Reiss et al. 1986), and Social Phobia Scale (Connor et al. 2000) that were assessed as part of a bigger research project.

**Table 1.** Comparison of the four word lists.

Variable	List 1 (*N* = 45)	List 2 (*N* = 45)	List 3 (*N* = 45)	List 4 (*N* = 45)	*F*-value (3,176)	*P*-value
Valence	5.21 (2.26)	5.24 (2.33)	5.35 (2.36)	5.43 (2.29)	0.08	.969
Arousal	3.99 (0.66)	4.07 (0.74)	3.93 (0.78)	4.13 (0.71)	0.71	.550
Self-relevance	5.95 (0.91)	6.09 (1.00)	6.15 (0.98)	6.24 (0.90)	0.13	.942
Concreteness	5.51 (1.11)	5.51 (1.14)	5.48 (1.23)	5.62 (1.24)	0.71	.551
Word length	9.91 (2.00)	9.96 (1.87)	9.96 (1.71)	9.71 (1.84)	0.18	.912
Word frequency	270 (368)	262 (307)	255 (348)	247 (363)	0.04	.991
Regularity	107 (240)	107 (173)	101 (208)	135 (255)	0.23	.877

*Note*. Standard deviations appear in parentheses below the means. Valence: 1 = highly negative, 5 = neutral, 9 = highly positive; Arousal: 1 = very low, 9 = very high; Concreteness: 1 = very concrete, 9 = very abstract. Word frequency is depicted per million.

### Procedure

**Figure 1.**
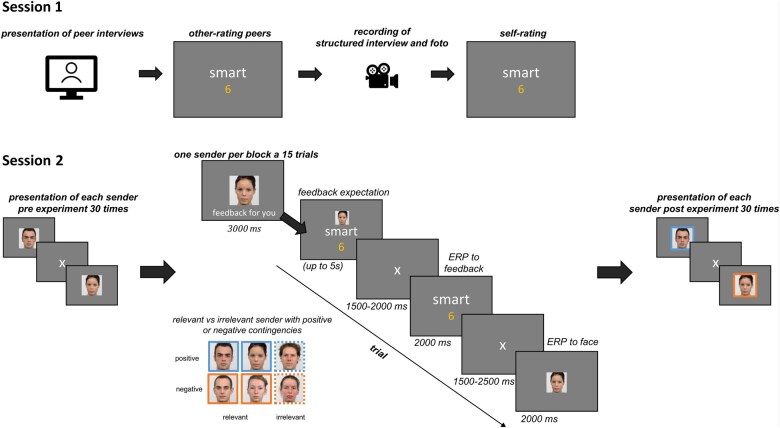
Experimental setup. (Session 1) The participants recorded a structured interview, and a photo of the participant was taken. Further, participants rated four supposedly other participants based on a video and performed a self-rating. (Session 2) The EEG data were recorded, with six sender faces presented before, during, and after the main experiment. Coloured frames were used for display but were not shown in the experiment. Two senders putatively gave feedback to someone else (self-irrelevant senders), highlighted with grey frames. An exemplary trial of the main experiment is shown at the bottom of the figure.

The study consisted of two sessions (cf Fig. 1). Both were conducted at the Institute of Medical Psychology. In the first session, after receiving information about the study and giving informed consent, the participant watched four videos in which supposedly other participants introduced themselves. Based on the videos, the participant provided feedback on 180 adjectives for the supposedly other participants, with values ranging from one (least applicable) to nine (most applicable). After providing the feedback, a self-introduction of the participant was recorded, and a picture of the supposedly later presentation was taken. The video lasted approximately three minutes, and five guideline questions were answered. Lastly, the participant rated him or herself using the same 180 adjectives (for the full list of adjectives, see Supplementary Section 4).

After 5–11 days, the participant returned for the second session. After being prepared for the EEG experiment and instructed about the upcoming feedback, participants were presented with photos of six senders (three male and three female faces; see Fig. 1) during the main experiment. In a familiarization run, each of the six faces was presented 30 times for 1000 ms before the main experiment, with an ITI varying between 1000 and 1500 ms, in a pseudo-randomized order, without repeating the same face in a row (see Fig. 1). The assignment of the different adjective lists and the faces were counterbalanced across participants to avoid associations with specific adjectives. Four senders (two male and two female) were instructed as having seen the participant’s video and provided ratings of the participant (relevant senders). Two senders (one male and one female) were instructed to be participants but provided feedback for someone else (irrelevant senders), serving as the ‘irrelevant-feedback’ control condition. Two relevant senders (one male and one female) were programmed to provide only positive feedback (i.e. more desirable ratings than the participants self-rating in session one), and two relevant senders (one male and one female) were programmed to provide only negative feedback (i.e. more undesirable ratings than the participants self-rating in session one; for each sender equal proportion of one, two, and three points deviation from the self-rating). Each sender gave feedback on 36 out of the 45 adjectives from the respective list. Trials/adjectives were selected where the respective sender could provide more positive or negative feedback, as compared to the participants’ self-ratings, i.e. all adjectives with extreme values (e.g. rated positive adjective nine out of nine) were excluded, and equally for the other senders. We, therefore, presented, on average, 200 out of the total of 216 trials (33 of 36 trials per sender).

In the main experiment, participants were instructed that a block with up to eighteen trials would be presented per sender, with the information about who is providing feedback and whether it is self-relevant or self-irrelevant at the beginning of each block (see Fig. 1). In each block, each trial started with the presentation of the sender’s face and trait adjective, after which participants needed to indicate their expectations (value between one and nine). Then, the feedback was presented for 2000 ms (value between one and nine). After the main experiment, participants had a self-paced break, and then all sender’s faces were represented 30 times for 1000 ms, with an ITI varying between 1000 and 1500 ms, in pseudo-randomized order, without repeating the same face in a row (see Fig. 1). After the experiment, participants were debriefed that no real feedback was provided. After the experiment, seventeen participants indicated doubts about the veridicality of the feedback upon request.

### EEG recording and preprocessing

EEG data were recorded from 64 BioSemi active electrodes using BioSemi’s Actiview software (version 8.11; www.biosemi.com). Additionally, four external electrodes measured horizontal and vertical eye movements. A Common Mode Sense active electrode (CMS) and a Driven Right Leg passive electrode (DLR) were used as ground electrodes. The offline data was preprocessed with BESA software (version 6.0; www.besa.de). The automatic eye-artifact correction was used to correct artefacts caused by eye movement, and low-quality channels were interpolated. The data was referenced to the average reference and filtered with a 0.1 Hz high-pass forward filter (6 dB/oct) and a 30 Hz low-pass zero-phase filter (24 dB/oct). The filtered data was segmented in epochs from 200 ms before feedback onset to 1500 ms after stimulus presentation, with a baseline correction from 100 ms before the stimulus. For ERP analyses, we examined the feedback processing for respective effects on the P1, N1/N170, EPN, FRN, and LPP components. We identified the P1 (75 to 95 ms), N1/N170 (130 to 170 ms), and EPN (270 to 370 ms) over symmetrical occipitotemporal sensors (six electrodes: TP7, P7, P9, TP8, P8, and P10) and the FRN (200 to 300 ms) over a frontocentral cluster (two electrodes: FCz, Fz). The LPP was identified between 400 and 1000 ms for feedback over an extended centro-parietal cluster (twenty electrodes: FC3, FC1, FCz, FC2, FC4, C3, C1, Cz, C2, C4, CP3, CP1, CPz, CP2, CP4, P3, P1, Pz, P2, P4). Analyses of the faces presented before, during, and after the main experiment are provided in the Supplementary material (see Supplementary Section 1).

### Statistical analysis

Statistical analyses were done using JASP, MatLab (www.mathworks.com, Version R2022b), and R Statistical Software (v4.4.2; R Core Team 2024). For feedback expectation ratings, for adjectives with a negative valence, the rating r was inverted (r' = 10-r), and r’ was used; thus, generally, high values corresponded to a high rating for positive and a low rating for negative adjectives. We tracked the progression of the difference between the expectations and the self-view, where negative values correspond to a more negative expectation and positive values, vice versa, to a more positive expectation. We used a linear mixed effects model (LME) analysis to investigate the effects of the sender self-relevance, sender valence, and trial repetition number of the feedback on the difference between the expectation and the initial self-view. The winning model was a full factorial model [expectation-self-view ∼ 1 + sender self-relevance * sender valence * trial repetition number + (1 + sender self-relevance * sender valence * trial repetition number |subject)], which included sender self-relevance, sender feedback valence, and trial repetition number as random intercepts and random slopes. We then calculated an ANOVA for the winning model. We report point estimates (*b*), 95% CI for LMMs, standard errors, *t*-values, and *P*-values for the fixed effects coefficients and point estimates (*b*) for the random effects (for random effects, see Supplementary Section 3). Exploratively and for illustration purpose we calculated a simplified model with trial repetition number recoded in d dichotomous variable [trial block: with early (i) vs. late (ii) repetition numbers] and calculated contrasts with the emmeans function (Lenth 2025).

For ERPs towards feedback incongruence with the self-view, a repeated measures ANOVA was calculated with the factor sender self-relevance (self-relevant feedback vs. self-irrelevant feedback) and sender valence (positive vs. negative) for each component. For the same feedback trials, we sorted conditions according to the participants’ expectations as exploratory analyses. For these analyses, two by three Repeated Measure ANOVAs were calculated with the factor’s sender self-relevance (self-relevant feedback vs. self-­irrelevant feedback) and feedback expectation (three levels: worse than expected, expected, and better than expected). While self-view incongruence was experimentally manipulated, trial numbers differed according to participants behaviour concerning feedback expectations. Partial eta-squared (η_P_^2^) was estimated to describe effect sizes where η_P_^2^ = 0.02 describes a small, η_P_^2^ = 0.13 a medium and η_P_^2^ = 0.26 a large effect (Cohen 1988). Post hoc comparisons used Holm’s correction for significant main effects for multiple comparisons. Degrees of freedom and corresponding *P*-values were corrected according to Greenhouse-Geisser correction if the Mauchly test violated the assumption of sphericity, while for readability, original degrees of freedom but corrected *P*-values and effect sizes are reported. The data that support the findings of this study have been deposited in the Open Science Framework (https://osf.io/6v7aq/). All data, participant information, and experimental design information are available in the respective repository. The study was not pre-registered.

## Results

### Behavioural data

#### Feedback expectation

We performed a LME model analysis to investigate the effects of the sender self-relevance, sender valence, and trial repetition number of the feedback on the difference of expectation and the self-rating. The ANOVA for the winning LME model revealed main effects of sender valence (*F*_(1,8015)_ = 85.53, *P* < .001), with more negative expectations for the negative senders (see Tables 2 and 3), and trial repetition number (*F*_(1,8015)_ = 45.98, *P* < .001). There was no main effect of sender self-relevance (*F*_(1,8015)_ = 0.74, *P* = .391). Further we found significant interaction effects between sender self-relevance and sender valence (*F*_(1,8015)_ = 4.83, *P* = .028), and sender valence and trial repetition number (*F*_(1,8015)_ = 74.21, *P* < .001), There were no significant interaction effects between sender self-relevance and trial repetition number (*F*_(1,8015)_ = 4.26, *P* = .039), and no three-way interaction between sender self-relevance and sender valence and repetition number (*F*_(1,8015)_ = .99, *P* = .319).

**Table 2.** Fixed effects of the linear mixed model.

Coefficient	Expectation—self-view						
	*b*	SE	*t*	df	*P*	(95% CI)	
						Lower	Upper
Intercept	−.941	.117	−8.033	8015	**<.001**	−1.171	−.711
Sender self-relevance (relevant vs. irrelevant)	−.151	.175	−.858	8015	.391	−.494	.193
**Sender valence (negative vs. positive)**	1.253	.135	9.248	8015	**<.001**	.987	1.518
**Repetition number**	−.014	.002	−6.781	8015	**<.001**	−.019	−.010
Sender self-relevance * Sender valence	.505	.230	2.198	8015	**.028**	.055	.956
Sender self-relevance * Repetition number	−.004	.007	−.542	8015	.588	−.018	.010
Sender valence * Repetition number	.025	.003	8.614	8015	**<.001**	.019	.031
Sender self-relevance * Sender valence * Repetition number	.011	.011	.997	8015	.319	−.011	.033
Model formula	Expectation-self-view ∼ 1 + sender self-relevance * sender valence * repetition number + (1 + sender self-relevance * sender valence * repetition number |subject)						

*Note*. Summary statistics show the overall average effects of sender self-relevance, sender valence, and trial repetition number. Significant effects are highlighted in bold font.

**Table 3.** Contrasts of the linear mixed model.

Contrast	Expectation—self-views			
	*b*	SE	*t*	*P*
Negative sender: relevant vs. irrelevant	−.026	.105	−.248	.801
**Positive sender: relevant vs. irrelevant**	−.305	.099	−3.08	**.002**
**Relevant sender: negative vs. positive**	−2.100	.099	−21.21	**<.001**
**Irrelevant sender: negative vs. positive**	−2.380	.125	−19.04	**<.001**
**Negative sender: early vs. late trial**	.273	.093	2.94	**.003**
**Positive sender: early vs. late trial**	−.256	.076	−3.37	**<.001**
Relevant sender: early vs. late trial	.070	.061	1.15	.251
Irrelevant sender: early vs. late trial	−.053	.098	−.541	.587
Model formula	Expectation-self-view ∼ 1 + sender self-relevance * sender valence * trial block + (1 + sender self-relevance * sender valence * trial block |subject)			

*Note*. Contrasts of the fixed effects coefficients for the sender self-relevance, sender valence, and trial block interactions computed from a simplified model with trial block for illustration purpose. Significant effects are highlighted in bold font.

**Figure 2.**
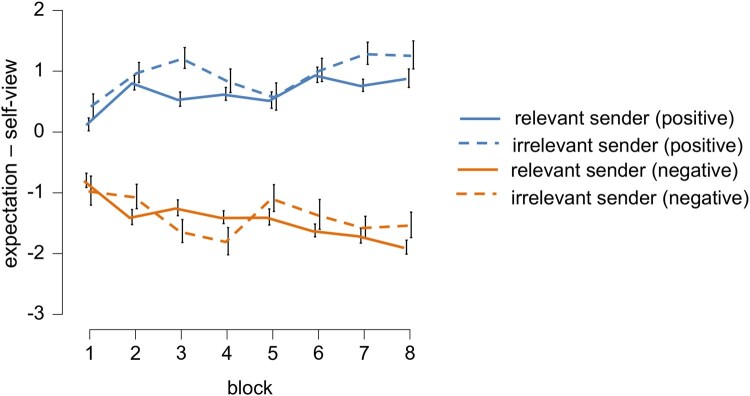
Difference between expectation and self-view over time for all sender types. Average differences between expectation and self-view ratings. For visualization reasons, we show eight blocks of averaged trials for positive and negative self-relevant senders (solid lines) and the respective self-irrelevant senders (dashed lines).

Concerning these interactions, unsurprisingly, sender valence differences increased over time, leading to more negative expectations for the negative senders and more positive expectations for positive senders (see Table 3, Fig. 2). Importantly, differences between the relevant and irrelevant positive senders were observed, and more positive expectations were held for the positive self-­irrelevant sender (see Table 3, Fig. 2). The individual coefficients for the random effects and explorative relationships between sender valence learning slopes and ERP differences between sender valences are reported in the Supplementary material (see Supplementary Sections 2 and 3).

### Event-related potential results

#### P1

For the P1, there was no main effect of feedback self-relevance (*F*_(1,39)_ = 0.12, *P* = .730, η_P_^2^ = .003) or sender valence (*F*_(1,39)_ = 1.06, *P* = .309, η_P_^2^ = .027) and no significant interaction between both (*F*_(1,39)_ = 1.09, *P* = .303, η_P_^2^ = .027).

Concerning feedback expectations, there was no main effect of sender self-relevance (*F*_(1,38)_ = 2.91, *P* = .096, η_P_^2^ = .071), a main effect of expectedness was observed (*F*_(2,76)_ = 3.58, *P* = .033, η_P_^2^ = .086), and no interaction between sender self-relevance and expectedness was found (*F*_(2,76)_ = 1.01, *P* = .368, η_P_^2^ = .026). P1 amplitudes were larger for worse feedback than for expected feedback (*t*_(38)_ = 2.66, *p*_holm_ = .029, Cohen’s *d* = 0.345). P1 amplitudes did not differ between better and expected feedback(*t*_(38)_ = 1.57, *p*_holm_ = .242, Cohen’s *d* = 0.203), or between worse and better feedback (*t*_(38)_ = 1.09, *p*_holm_ = .278, Cohen’s *d* = 0.141).

#### N1/N170

For the N1/N170, likewise, there was no main effect of self-relevance (*F*_(1,39)_ = 0.03, *P* = .866, η_P_^2^ < .001) or sender valence (*F*_(1,39)_ = 0.92, *P* = .344, η_P_^2^ = .023) and no significant interaction between both (*F*_(1,39)_ < 0.01, *P* = .991, η_P_^2^ < .001).

Concerning feedback expectations, there were no main effects of sender self-relevance (*F*_(1,38)_ = 1.02, *P* = .320, η_P_^2^ = .026) and expectedness (*F*_(2,76)*_ = 0.88, *P* = .399, η_P_^2^ = .023), and no interaction between sender self-relevance and expectedness was found (*F*_(2,76)_ = 2.82, *P* = .066, η_P_^2^ = .069).

#### Early posterior negativity

**Figure 3.**
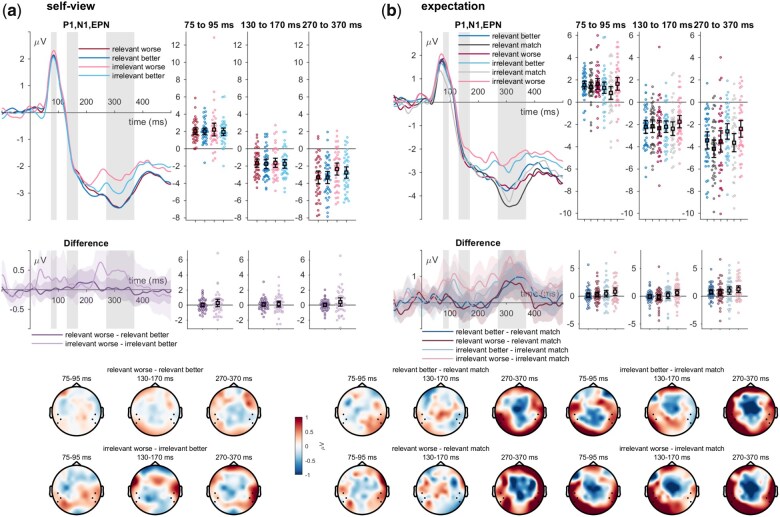
P1, N1/N170, and EPN effects of feedback incongruence with the (a) self-view and (b) expectation. ERP waveforms show the time course for worse (red/pink), congruent (dark/light grey), and better feedback (dark/light blue lines) for the ‘relevant’ and ‘irrelevant’ senders. Error bars show 95% CIs. Difference plots contain 95% bootstrap confidence intervals of intra-individual differences. Scalp topographies below depict the amplitude differences for the worse/better feedback and the congruent/expected feedback.

For the EPN, a main effect of self-relevance was found (*F*_(1,39)_ = 24.68, *P* < .001, η_P_^2^ = .388; see Fig. 3), with more negative amplitudes for the relevant senders (*t*_(39)_ = −4.97, *p*_holm_ < .001, Cohen’s *d* = −0.347). There was no main effect or valence (*F*_(1,39)_ = 1.48, *P* = .231, η_P_^2^ = .037) and no significant interaction between sender self-relevance and valence (*F*_(1,39)_ = 1.41, *P* = .242, η_P_^2^ = .035).

Concerning feedback expectations, there were both main effects of sender self-relevance (*F*_(1,38)_ = 17.78, *P* < .001, η_P_^2^ = .319), and expectedness (*F*_(2,76)_ = 13.79, *P* < .001, η_P_^2^ = .266), while no interaction between sender self-relevance and expectedness was found (*F*_(2,76)_ = 0.98, *P* = .380, η_P_^2^ = .025). EPN amplitudes were more negative-­going for feedback from relevant senders (*t*_(38)_ = −4.22, *p*_holm_ < .001, Cohen’s *d* = −0.321) and for expected as compared to both worse (*t*_(38)_ = −4.71, *p*_holm_ < .00<, Cohen’s *d* = −0.368) and better feedback(*t*_(38)_ = −4.37, *p*_holm_ < .001, Cohen’s *d* = −0.341) with no differences between worse and better feedback (*t*_(38)_ = 0.34, *p*_holm_ = .735, Cohen’s *d* = 0.027).

#### Feedback-related negativity

**Figure 4.**
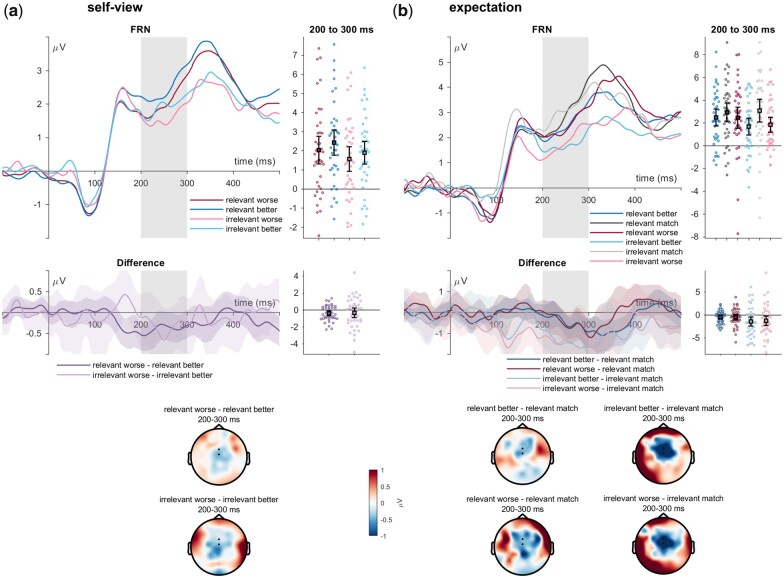
FRN effects of feedback incongruence with the (a) self-view and (b) expectation. ERP waveforms show the time course for worse (red/pink), congruent (dark/light grey), and better feedback (dark/light blue lines) for the ‘relevant’ and ‘irrelevant’ senders. Error bars show 95% CIs. Difference plots contain 95% bootstrap confidence intervals of intra-individual differences. Scalp topographies below depict the amplitude differences for the worse/better feedback and the congruent/expected feedback.

For the FRN, a main effect of self-relevance was found (*F*_(1,39)_ = 6.92, *P* = .012, η_P_^2^ = .151; see Fig. 4a), with more negative amplitudes for the irrelevant senders (*t*_(39)_ = −2.63, *p*_holm_ = .012, Cohen’s *d* = −0.241). There was a main effect of sender valence (*F*_(1,39)_ = 5.37, *P* = .026, η_P_^2^ = .121), with more negative amplitudes for the negative senders (*t*_(39)_ = −2.32, *p*_holm_ = .026, Cohen’s *d* = −0.159). There was no significant interaction between self-relevance and valence (*F*_(1,39)_ = 0.01, *P* = .910, η_P_^2^ < .001).

**Figure 5.**
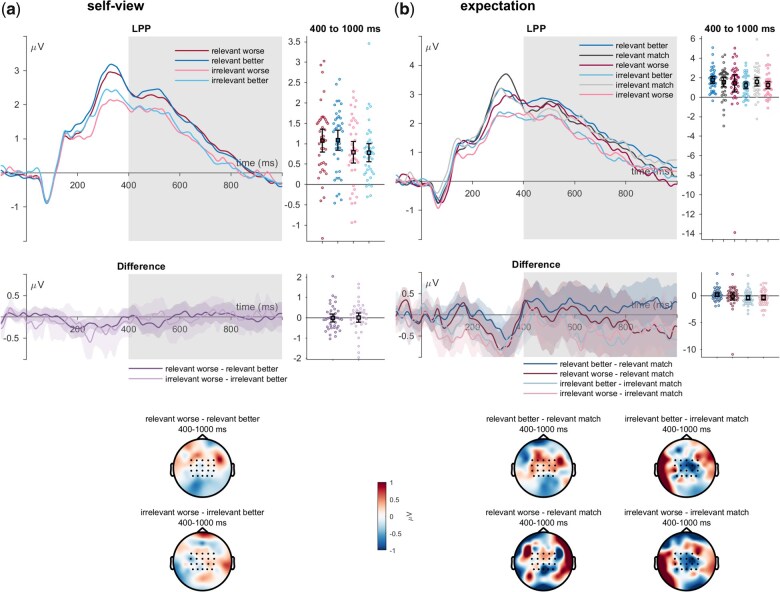
LPP effects of feedback incongruence with the (a) self-view and (b) expectation. ERP waveforms show the time course for worse (red/pink), congruent (dark/light grey), and better feedback (dark/light blue lines) for the ‘relevant’ and ‘irrelevant’ senders. Error bars show 95% CIs. Difference plots contain 95% bootstrap confidence intervals of intra-individual differences. Scalp topographies below depict the amplitude differences for the worse/better feedback and the congruent/expected feedback.

Concerning feedback expectations, there was no main effect of sender self-relevance (*F*_(1,38)_ = 2.54, *P* = .119, η_P_^2^ = .063), a main effect of expectedness was observed (*F*_(2,76)_ = 7.31, *P* = .001, η_P_^2^ = .161), and no interaction between sender and expectedness was found (*F*_(2,76)_ = 1.66, *P* = .197, η_P_^2^ = .042). FRN amplitudes were more negative-going for worse feedback than for expected feedback (*t*_(38)_ = −3.17, *p*_holm_ = .004, Cohen’s *d* = −0.321) and for better feedback than for expected feedback(*t*_(38)_ = −3.43, *p*_holm_ = .003, Cohen’s *d* = −0.347) with no differences between worse and better feedback (*t*_(38)_ = 0.26, *p*_holm_ = .794, Cohen’s *d* = 0.026).

#### Late positive potential

For the LPP, a main effect of self-relevance was found (*F*_(1,39)_ = 9.85, *P* = .003, η_P_^2^ = .202; see Fig. 5), with more positive LPP amplitudes for the relevant senders (*t*_(39)_ = 3.14, *p*_holm_ = .003, Cohen’s *d* = 0.371). There was no main effect or sender valence (*F*_(1,39)_ = 0.08, *P* = .785, η_P_^2^ = .002) and no significant interaction between sender self­relevance and valence (*F*_(1,39)_ = 0.11, *P* = .745, η_P_^2^ = .003).

Concerning feedback expectations, there were no main effects of sender (*F*_(1,38)_ = 0.91, *P* = .345, η_P_^2^ = .023), or of expectedness (*F*_(2,76)_ = 0.89, *P* = .416, η_P_^2^ = .023), and there was also no interaction between sender self-relevance and expectedness (*F*_(2,76)*_ = 1.09, *P* = .326, η_P_^2^ = .028).

## Discussion

This study used a realistic social interaction design to study the effects of positive and negative social evaluative feedback from relevant and irrelevant senders on behavioural and neuronal measures. Social feedback was varied regarding self-relevance and valence, the latter based on the incongruence with the self-ratings from the first session. We tested how learning about the sender’s behaviour changed feedback expectations throughout the experiment and examined ERPs towards the feedback incongruence with both the self-view and the feedback expectation. Participants’ expectation updating was examined only on their behavioural responses, which showed over time, the differentiation between the positive and negative senders. However, contrary to our expectations, we observed that participants updated their expectations to a larger extent for the negative senders and towards the positive self-irrelevant as compared to the self-relevant senders. We observed expected increases of the EPN and LPP amplitudes for self-relevant compared to self-irrelevant feedback with no valence differences. Such valence differences were observed on the FRN, with a larger negativity for the negative senders. Here, analyses of incongruence with the feedback expectation also showed a larger negativity for both worse and better feedback than for expected feedback.

Concerning the difference between feedback expectation and self-view, participants adjusted their expectations over time. The LME analysis shows that the full factorial model with random slopes describes the data best, showing large effects of sender valence that increased with trial repetitions. We also found that participant changed their expectations to a larger extent towards the negative senders, which is in contrast to our expectations. People are reasoned to update their expectations towards positive information asymmetrically (Hepper et al. 2011) and are supposed to incorporate unexpected positive feedback better than negative feedback (Kube et al. 2020). Still, these theoretical accounts and the empirical evidence are based on updating beliefs about themselves (Korn et al. 2012), their personal risks (Garrett et al. 2014), or their abilities (Kube et al. 2019). To explain the unexpected findings, in the current study, participants were required to predict the upcoming feedback without being asked to judge the subjective validity or measure self-related consequences. Other aspects might be that, while we only selected traits where more negative or positive feedback was possible, due to the positively skewed self-ratings (average 6.33 on a scale from one to nine), participants could deviate more towards the negative than the positive direction. Furthermore, especially for the self-relevant positive senders, social desirability biases might also lower the readiness to indicate highly positive values to avoid being seen as arrogant or narcissistic.

Another aspect that might explain the specifically better learning for the self-irrelevant positive senders could be the lack of personal involvement, where the feedback prediction might exhibit more similarity to a performance task, which is more rewarding when being able to predict a specific feedback value correctly, and less influenced by biases of the self-view (Müller-Pinzler et al. 2019). Concerning the negative self-related senders, as pointed out in the predictive processing account (Kube et al. 2020; see also Pinquart et al. 2021), cognitive immunization strategies are expected to question, e.g. the feedback’s veridicality or the sender’s competence (Kube et al. 2020; see also Pinquart et al. 2021). In this vein, we have a larger proportion of participants questioning the veridicality of the feedback (seventeen; see also the limitation section below), which may also be based on the clarity of sender behaviour (i.e. always providing better or worse feedback). As noted above, positively biased learning may be observed in ratings of the sender’s competence, likability, and participants’ self-reported mood or self-view changes. Future studies may use such measures to clarify and disentangle the effects of sender valence on feedback expectation and self-integration processes.

Concerning ERP effects of feedback incongruence with the self-view and feedback expectations, we found no significant effects of the self-view incongruence of feedback on the P1 and the N1/N170 amplitudes. Concerning ERP effects of incongruence with the feedback expectations, a main effect of expectedness for P1 amplitudes was observed with larger amplitudes for worse-than-expected feedback. The P1 modulations play a role in attention allocation (Klimesch et al. 2007, Slagter et al. 2016), and studies frequently show increased P1 amplitudes towards threat (Brown et al. 2010, Gupta et al. 2019) and, similarly, increased P1 amplitudes in anticipation of both negative and more relevant social evaluative feedback (Schindler et al. 2019b). These findings are largely in line with the observation that early effects are variable (Peters et al. 2024), with studies reporting opposing or no effects of the feedback valence or self-relevance (e.g. see Leitner et al. 2014, Schindler et al. 2020, 2021, Weik et al. 2022). Concerning later stages, we find the main effect of self-relevance on the EPN, FRN, and LPP. The increased negativity during the EPN and positivity during the LPP for the more relevant senders align with the literature and may represent an overall attributed importance of the feedback (Peters et al. 2024), similar to many previous studies (e.g. see Rohr and Abdel Rahman 2015, Schindler et al. 2015, Schindler and Kissler 2016a, 2018; see also Peters et al. 2024). It is important to note that our study did not include a neutral or even a congruent feedback condition, for which (incongruent) positive and negative feedback has shown reliably and repeatedly increased EPN and LPP amplitudes (Peters et al. 2025, for review, see Peters et al. 2024). While the EPN is typically interpreted in terms of early responses to emotionally relevant or arousing information, the late increase in amplitudes is typically interpreted as an elaboration of stimulus, encoding, and updating (Dolcos and Cabeza 2002, Schindler et al. 2022).

On the contrary, on the FRN, we observed valence effects for the senders when only considering the incongruence with the self-view, and we also observed the effects of feedback expectation incongruence. The FRN amplitudes were more negative for the negative senders. When sorting trials only according to the feedback expectations, losing the sender valence information, specific effects of expectation violation by valence (i.e. worse vs. better feedback than expected) were not observed. Here, FRN amplitudes were generally increased for all unexpected feedback compared to expected feedback, in line with the majority of findings in studies on social evaluative feedback processing (Peters et al. 2024). Effects of the EPN and the FRN were found to be highly similar but mirrored. Note that for both ERPs, a larger negativity is typically interpreted in terms of increased attentional selection and subsequent processing of the stimulus. Finally, we explored correlations between individual sender valence learning slopes and average sender expectation ratings to the respective ERP effects, showing for the latter relationships with the P1 and FRN that may stimulate further research questions (see Supplementary Section 2).

### Constraints of generalizability and outlook

While we expected sender attribution differences by their feedback self-relevance and through constant negative or positive feedback behaviour, we did not vary other sender attributes (e.g. expertise, relatedness), which are suggested to impact learning and updating effects (Collins and Stukas 2006; see also Falk and Scholz 2018, Schindler et al. 2019a). We solely focused on expectation changes and did not measure the changes in mood, sender ratings, self-view, or the intention to interact. More research has yet to investigate what consequences the adjustment of expectations in a social interaction has. One likely consequence if participants can predict how others like or dislike them could be the future intention to interact and thus approach or avoid the senders (Schoch et al. 2015, Nikitin and Schoch 2021). In addition, we explored possible changes in ERPs to the supposed faces of senders according to their self-relevance and observed little changes (see Supplementary Section 1). This may be due to the number of repetitions per face (∼100 times) since we aimed to induce sufficient learning experience per sender (face). Future studies could use more sender faces by facilitating learning through either very salient behaviour or instructed person knowledge (e.g. see Suess et al. 2015, Baum et al. 2020). Finally, we selected only trait adjectives, where senders provided better or worse feedback than the initial self-rating. This increased learning about sender behaviour but at the same time reduced the credibility, given that a larger number than usual (seventeen) indicated doubts about the feedback veridicality after the second third of the experiment.

## Conclusion

The current study tested the effects of social evaluative feedback on updating the feedback expectations through learning about the sender’s behaviour. We found that participants adjusted their feedback expectations for positive and negative senders over time while changing expectations stronger towards self-irrelevant positive and generally towards negative feedback senders. An assumed positively biased self-related updating does apply to the feedback expectation ratings of this study and may be bound to more self-related evaluations. Early neuronal responses to the feedback showed an increased P1 when being confronted with worse-than-expected feedback. Self-relevance of senders generally increased the EPN and LPP amplitudes, irrespective of the sender valence. In contrast, the FRN was increased by negative sender valence, but the sorting trial according to the participants’ expectations showed only an effect of expectedness, irrespective of the valence violation. The results demonstrate a specific impact of sender attributions on feedback predictions and ERPs towards the feedback.

## Supplementary Material

nsaf062_Supplementary_Data

## Data Availability

The data that support the findings of this study have been deposited in the Open Science Framework (https://osf.io/6v7aq/). All data, participant information, and experimental design information are available in the respective repository. The study was not pre-registered.
