# Supplementary material for: Differential effects of social feedback valence and self-relevance on brain responses and behaviour
Source: Soc Cogn Affect Neurosci. 2025 Jun 20;20(1):nsaf062. doi: 10.1093/scan/nsaf062 (PMC12419807; doi:10.1093/scan/nsaf062)
Supplement: nsaf062_Supplementary_Data [file nsaf062_supplementary_data.doc]

**Differential effects of social feedback valence and self-relevance on brain responses and behavior**

**SUPPLEMENTARY MATERIALS**

Hanne Helming^1^, Antje Peters^1,2^, Su Arkun^1^, Maximilian Bruchmann^1,2^, Robert Moeck^1^, Thomas Straube^1,2^, and Sebastian Schindler^1,2^*

^1^Instutite for Medical Psychology and Systems Neuroscience, University of Münster, Germany

^2^ Otto Creutzfeldt Center for Cognitive and Behavioral Neuroscience, University of Muenster

* Corresponding author

**Correspondence addresses**

University of Münster

Institute of Medical Psychology and Systems Neuroscience

Von-Esmarch Straße 52, 48149 Münster

E-mail: Sebastian.Schindler@ukmuenster.de

## ERPs to the social evaluative feedback sender faces

- 1. **Background**

In addition to the processing of social evaluative feedback and its impact on changes in feedback expectations, we examined ERPs to the supposed sender faces. Social evaluative feedback may also change the emotional meaning of the sender's face. Recent studies showed that faces associated with negative social information do not differ at the stage of the P1 (e.g., see Luo et al., 2016; Schindler et al., 2021) but may elicit increased N170 (see Baum & Abdel Rahman, 2021; Schindler et al., 2021; Luo et al., 2016; Giménez-Fernández et al., 2020; but see Xu et al., 2016). The N170 degree has been suggested to depend on the extent of learning (Schindler et al., 2023). However, until today, only one study directly tested ERPs towards faces after social evaluative feedback. The study used the faces of many supposed senders that provided binary ('like' or 'dislike') feedback and focused on the N170 component, for which increased amplitudes for faces associated with negative feedback were found (Qi et al., 2017). From other approaches, studies show that evaluative social person knowledge can increase EPN and/or LPP responses (Abdel Rahman, 2011; Suess et al., 2015; Schindler et al., 2021b; Xu et al., 2016; Luo et al., 2016; Krasowski et al., 2021; Baum and Abdel Rahman, 2021; but see no EPN effects in Baum et al., 2018; Kissler and Strehlow, 2017; see no LPP effects in Luo et al., 2016). It may be that receiving self-relevant social evaluative feedback changes the neuronal responses the sender faces. Thus, we tested ERPs towards inherently neutral faces that acquired social relevance and valence throughout learning during the main experiment across the whole sequence of ERPs from early to late processing stages.

- 1. **Methods**

For ERP analyses, four participants had bad EEG data for the pre- or post-experimental face presentation run. Therefore, we here examined only 36 participants for their ERPs towards the faces on all three face presentation runs. We tested the effects on the P1, N170, EPN, and LPP components. We identified the P1 (90 to 110 ms), N170 (120 to 170 ms), and EPN (170 to 270 ms) over symmetrical occipitotemporal sensors (six electrodes: TP7, P7, P9, TP8, P8, P10) and the LPP (400 to 1000 ms) over parietal regions (eight electrodes: P3, P1, Pz, P2, P4, PO3, POz, PO4).

Statistical analyses used three (factor time point: pre-experimental, main experiment, post-experimental presentation) by two (factor self-relevance: self-relevant vs. self-irrelevant) by two (factor valence: positive vs. negative) Repeated Measures ANOVAs for ERPs of interest. Partial eta-squared (η_P_^2^) was estimated to describe effect sizes where η_P_^2^ = 0.02 describes a small, η_P_^2^ = 0.13 a medium and η_P_^2^ = 0.26 a large effect (Cohen, 1988). Post hoc comparisons used Holm's correction for significant main effects for multiple comparisons. Degrees of freedom and corresponding p-values were corrected according to Greenhouse-Geisser correction if the Mauchly test violated the assumption of sphericity, while for readability, original degrees of freedom but corrected p-values and effect sizes are reported.

**1.3. Results**

## P1

For the P1 towards faces, there was a main effect of time (*F*_(2,70)_ = 4.31, *p =* .017, η_P_² = .110, see Supplementary Figure S1), with reduced P1 amplitudes during the main experiment compared to pre- (*t*_(35)_ = -2.90, *p*_holm_ = .015, Cohen's d = -0.241) but not compared to the post-experimental presentation run (*t*_(35)_ = -1.82, *p*_holm_ = .146, Cohen's d = -0.151), and not differing between pre- and post-experimental presentation (*t*_(35)_ = 1.08, *p*_holm_ = .282, Cohen's d = 0.090). There were no main effects of the sender-face self-relevance (*F*_(1,35)_ = 2.73, *p =* .108, η_P_² = .072) and of the sender-face valence (*F*_(1,35)_ < 0.01, *p =* .971, η_P_² < .001). There were no significant interactions between the sender-face self-relevance and valence (*F*_(1,35)_ = 2.88, *p =* .099, η_P_² = .076), sender-face self-relevance and time (*F*_(2,70)_ = 1.03, *p =* .364, η_P_² = .028), sender-face valence and time(*F*_(2,70)_ = 0.45, *p =* .643, η_P_² = .013), and no three-way interaction (*F*_(2,70)_ = 0.07, *p =* .935, η_P_² = .002).

## N170

For the N170 towards faces, there was a main effect of time (*F*_(2,70)*_ = 53.06, *p <* .001, η_P_² = .603, see Supplementary Figure S1), with increased N170 amplitudes during the main experiment compared to pre- (*t*_(35)_ = -9.35, *p*_holm_ < .001, Cohen's d = -0.735) and compared to the post-experimental presentation run (*t*_(35)_ = -8.42, *p*_holm_ < .001, Cohen's d = -0.662), not differing between pre- and post-experimental presentation (*t*_(35)_ = 0.93, *p*_holm_ = .354, Cohen's d = 0.073). There were no main effects of the sender-face self-relevance (*F*_(1,35)_ = 2.31, *p =* .138, η_P_² = .062) and of the sender-face valence (*F*_(1,35)_ = 0.03, *p =* .872, η_P_² < .001). There were no significant interactions between the sender-face self-relevance and valence (*F*_(1,35)_ = 0.34, *p =* .564, η_P_² = .010), sender-face self-relevance and time (*F*_(1,35)_ = 1.77, *p =* .178, η_P_² = .048), sender-face valence and time(*F*_(2,70)_ = 0.37, *p =* .690, η_P_² = .011), and no three-way interaction (*F*_(2,70)_ = 0.09, *p =* .914, η_P_² = .003).


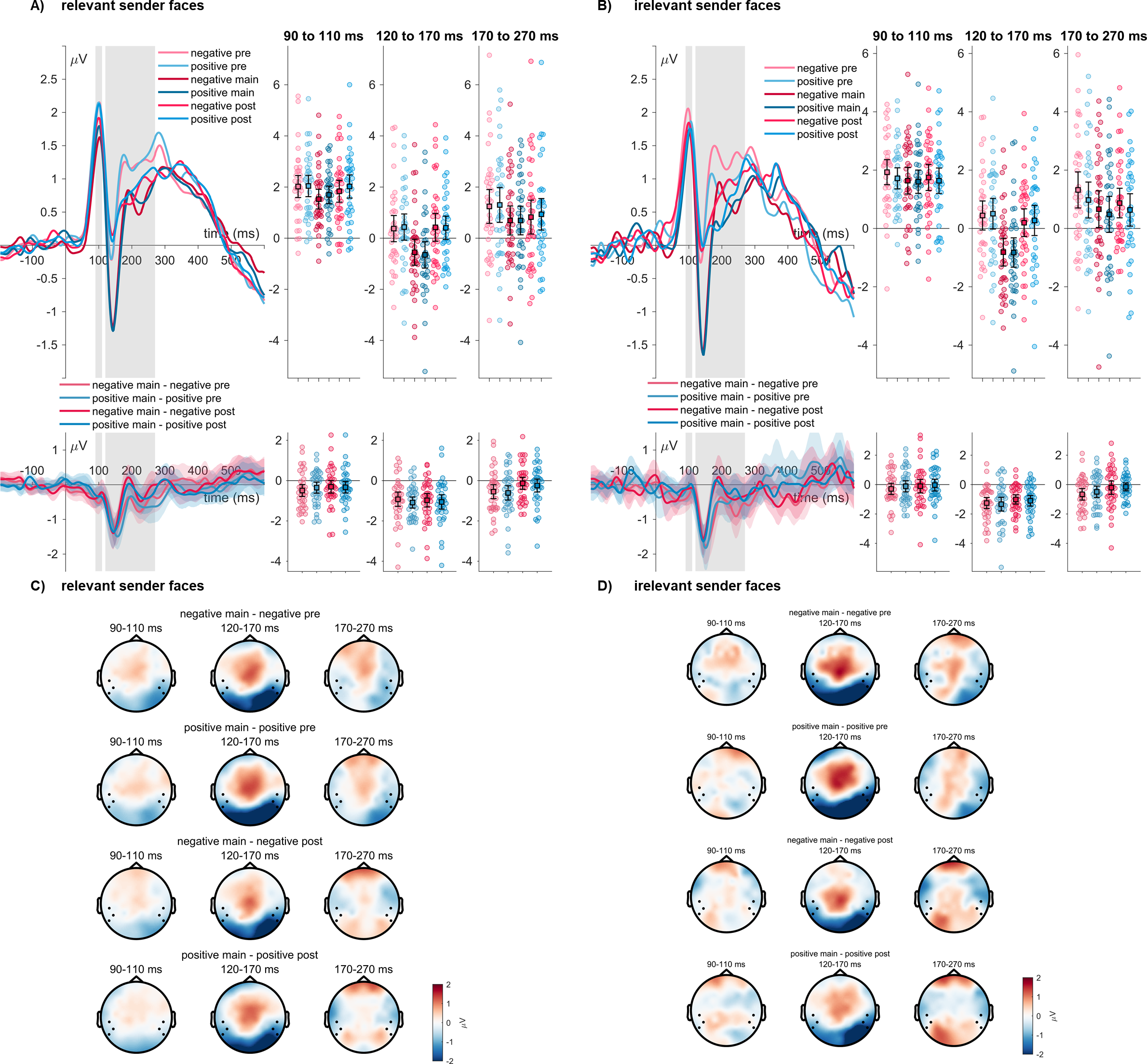


**Supplementary Figure S1. P1, N170, and EPN effects of time, sender self-relevance, and sender-valence. A)** ERP waveforms for self-relevant and **B)** self-irrelevant positive (turquoise, dark blue, light blue) and negative faces (pink, red, light red) show the time course for faces presented before the main experiment ('pre'), during the main experiment after feedback ('main') and after the main experiment ('post'). Error bars show 95% confidence intervals. Difference plots contain 95% bootstrap confidence intervals of intra-individual differences. **C)** and **D)** Scalp topographies below depict the amplitude differences between the main experiment and presentation before and after the experiment for four different face conditions.

## *EPN*

For the EPN towards faces, there was a main effect of time (*F*_(2,70)_ = 9.62, *p <* .001, η_P_² = .216, see Supplementary Figure S1), with increased EPN amplitudes during the main experiment compared to pre- (*t*_(35)_ = -4.30, *p*_holm_ < .001, Cohen's d = -0.306) but not compared to the post-experimental presentation run (*t*_(35)_ = -1.38, *p*_holm_ = .173, Cohen's d = -0.098). Further, faces presented post-experimentally led to an increased EPN compared to the pre-experimental presentation (*t*_(35)_ = -2.92, *p*_holm_ = .009, Cohen's d = 0.208). There were no main effects of the sender-face self-relevance (*F*_(1,35)_ = 2.60, *p =* .116, η_P_² = .069) and of the sender-face valence (*F*_(1,35)_ = 1.56, *p =* .219, η_P_² = .043). There were no significant interactions between the sender-face self-relevance and valence (*F*_(1,35)_ = 3.03, *p =* .091, η_P_² = .080), sender-face self-relevance and time (*F*_(1,35)_ < 0.01, *p =* .999, η_P_² < .001), sender-face valence and time(*F*_(2,70)_ = 0.20, *p =* .817, η_P_² = .006), and no three-way interaction (*F*_(2,70)_ = 0.34, *p =* .711, η_P_² = .010).

***LPP***

For the LPP towards faces, there was a main effect of time (*F*_(2,70)_ = 10.48, *p <* .001, η_P_² = .230; see Supplementary Figure S2), with reduced LPP amplitudes during the main experiment compared to pre- (*t*_(35)_ = -4.54, *p*_holm_ < .001, Cohen's d = -0.499) and compared to the post-experimental presentation run (*t*_(35)_ = -2.77, *p*_holm_ = .014, Cohen's d = -0.304). There were no differences between faces presented post-experimentally and the pre-experimental presentation (*t*_(35)_ = -1.78, *p*_holm_ = .080, Cohen's d = 0.195). There was a main effect of the sender-face self-relevance (*F*_(1,35)_ = 4.36, *p =* .044, η_P_² = .111), with a generally larger LPP for self-relevant faces (*t*_(35)_ = 2.09, *p*_holm_ = .044, Cohen's d = 0.192). There was no main effect on the sender-face valence (*F*_(1,35)_ = 0.16, *p =* .694, η_P_² = .004). There were no significant interactions between the sender-face self-relevance and valence (*F*_(1,35)_ < 0.01, *p =* .939, η_P_² < .001), sender-face self-relevance and time (*F*_(1,35)_ = 0.88, *p =* .421, η_P_² = .024), sender-face valence and time(*F*_(1,35)_ = 1.58, *p =* .213, η_P_² = .043), and no three-way interaction (*F*_(2,70)_ = 0.31, *p =* .733, η_P_² = .009).

Although there was no interaction between self-relevance and time, we explored the main effect for all time points. We observed no main effect of self-relevance pre-experimentally (*F*_(1,35)_ = 0.06, *p =* .809, η_P_² = .002), but a larger LPP for self-relevant faces during the main experiment (*F*_(1,35)_ = 5.19, *p =* .029, η_P_² = .129), being not statistically significant at the post-experimental presentation (*F*_(1,35)_ = 2.96, *p =* .094, η_P_² = .078).

**
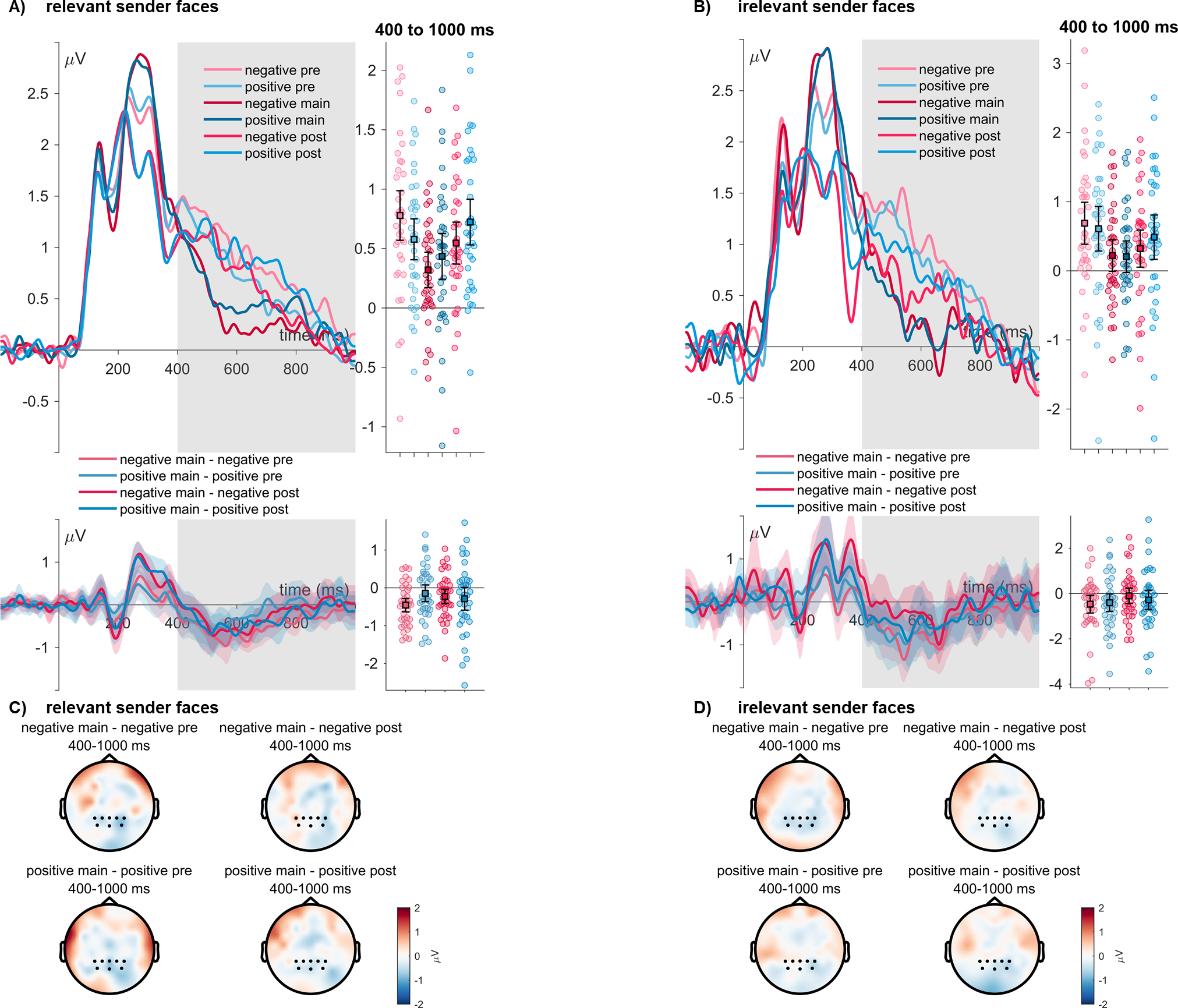
**

**Supplementary Figure S2. LPP effects of time, sender self-relevance, and sender-valence. A)** ERP waveforms for self-relevant and **B)** self-irrelevant positive (turquoise, dark blue, light blue) and negative faces (pink, red, light red) show the time course for faces presented before the main experiment ('pre'), during the main experiment after feedback ('main') and after the main experiment ('post'). Error bars show 95% confidence intervals. Difference plots contain 95% bootstrap confidence intervals of intra-individual differences. **C)** and **D)** Scalp topographies below depict the amplitude differences between the main experiment and presentation before and after the experiment for four different face conditions.

**1.4. Discussion**

In addition to the effects of social evaluative feedback on neuronal responses and behavioral changes in feedback expectations, we examined how receiving self-relevant social evaluative feedback changes neuronal responses to the sender's faces. We manipulated both the self-relevance and the valence of faces by having sender faces that provided either only positive or negative social evaluative feedback. Using the full-factorial analyses over time, we observed on all ERPs mainly effects of time, with reduced P1 and LPP amplitudes but increased N170 and EPN amplitudes for faces following the social evaluative feedback in the main experiment. It is worth noting that these main effects of time should be interpreted cautiously since the characteristics of the context can influence modulations. During the main experiment, both the preceding social evaluative feedback may have induced overlapping processes lasting into the face onset as well as a larger temporal delay between each face presentation. Still, interestingly, exposure to faces during the main experiment seems to have lasting effects on ERPs since EPN amplitudes remained more negative after the experiment than they were in the pre-experimental run, although here, the presentation parameters were identical.

We found almost no effects of the self-relevance or valence of the faces. Descriptively, N170 amplitudes towards self-irrelevant faces did change with a larger magnitude from the pre-experimental presentation to the main experiment. This contrasts our assumption of larger learning about self-relevant faces, similar to changes in expectations (see the main manuscript). It may be that the larger degree of learning can be related to the changes in N170 amplitudes. A previous review pointed out that N170 modulations based on evaluative learning depend on the extent of learning (Schindler et al., 2023), which in this study was stronger for self-irrelevant feedback. It might be that different feedback processing and integration processes may have overlapped or conflicted with each other, such as updating the prediction of the sender's behavior, updating the Valences toward the sender, and updating/changes toward the sender's face. We could not observe specific effects of negative social evaluative sender valence. This has been reported in one study for faces providing global 'reject' as compared to 'accept' feedback (Qi et al., 2017). While we could not show such an effect for the N170, this previous study relied on the presentation of many different identities for senders (Qi et al., 2017), and thus, a possible gain for single-trial learning of negative feedback cannot be tested in our study with only a few senders and intense learning about their behavior.

We also a main effect of self-relevance with increased late ERPs for self-relevant faces. While we observed no interaction with time, we explored this difference for all three presentation runs. While no differences were present before the main experiment, these became significant in the main experiment, being reduced and not statistically significant after the experiment. While LPP amplitudes were decreased during the main experiment, these relatively increased LPP amplitudes may reflect differences in late elaborative attention processes towards the self-relevant faces. This relative difference also seems descriptively largest for senders of self-relevant positive feedback, which elicited the highest positivity. This aligns with ideas that during the LPP, different elaborative processes, such as explicit emotional appraisal, self-referential processing, and information integration occur (e.g., see Dolcos & Cabeza, 2002; Hajcak et al., 2010), and studies have also proposed a bias for encoding positive and self-relevant information (e.g., see Xie et al., 2021), and positive social evaluative feedback enhancing the interest in the sender (Vanderhasselt et al., 2015) and likelihood of reciprocal positive behavior (Kroczek & Mühlberger, 2022).

## Relationships between expectation updating / average expectations per sender and ERPs towards sender feedback

To relate the individual updating coefficient of the feedback valence to the ERPs, we run a number of explorative correlational tests. We correlated the individual updating coefficient of sender valence with the ERP difference of negative and positive senders for their feedback, using two-sided Pearson correlations for all ERPs of interest (P1, N1/N170, EPN, LPP). We found no correlation of the ERP differences between the positive and negative self-incongruent feedback senders and individual updating coefficient of sender valence (P1: Pearson *r* = .076, *p =* .644; N1/N170: Pearson *r* = -.138, *p =* .402; EPN: Pearson *r* = -.056, *p =* .734; FRN: Pearson *r* = -.025, *p =* .880; LPP: Pearson *r* = .019, *p =* .907).

We also related the average expectation ratings (as for all analyses calculated as the rating differences to the self-view, where negative values indicate more negative expectations) for each sender with the average ERPs towards feedback from the respective senders and sender faces, using two-sided Pearson correlations for all ERPs of interest (P1, N1/N170, EPN, LPP). We observed for the P1 a reduced amplitude when participants expected more positive feedback or increased P1 when participants expected more negative feedback from relevant senders only (P1: relevant negative Pearson *r* = -.363, *p =* .021; relevant positive Pearson *r* = -.391, *p =* .013; irrelevant negative Pearson *r* = -.090, *p =* .582; irrelevant positive Pearson *r* = -.209, *p =* .195). Concerning the N1/N170, no significant relationships were observed between average expectation ratings and the respective ERPs towards the feedback (N1/N170: relevant negative Pearson *r* = -.091, *p =* .576; relevant positive Pearson *r* = -.206, *p =* .201; irrelevant negative Pearson *r* = .200 *p =* .216; irrelevant positive Pearson *r* = .005, *p =* .974). Likewise, for the EPN, no significant relationships were observed (EPN: relevant negative Pearson *r* = -.005, *p =* .973; relevant positive Pearson *r* = -.273, *p =* .089; irrelevant negative Pearson *r* = .134 *p =* .410; irrelevant positive Pearson *r* = -.034, *p =* .834), as well as for the LPP (LPP: relevant negative Pearson *r* = -.012, *p =* .940; relevant positive Pearson *r* = .088, *p =* .590; irrelevant negative Pearson *r* = .009 *p =* .955; irrelevant positive Pearson *r* = -.146, *p =* .370). In contrast, for the FRN, a positive relationship was found for the relevant positive sender, showing that more positive expectations led to a more positive going amplitude (FRN: relevant negative Pearson *r* = .099, *p =* .544; relevant positive Pearson *r* = .325, *p =* .041; irrelevant negative Pearson *r* = -.024 *p =* .882; irrelevant positive Pearson *r* = -.041, *p =* .800).

To summarize, we observed only a few significant correlations that should be interpreted with care, given the number of explored correlations. We observed no relationship of the differential learning slopes of sender valence and ERP differences between these senders. For average ratings, we found that those participants who expected more negative feedback from relevant senders were showing increased P1 amplitudes, similar to analyses of expectation ratings in the main manuscript. Here, we discussed that increased P1 amplitudes toward threat are observed (Brown et al., 2010; Gupta et al., 2019). As we related individual average expectations and ERPs, this might reflect individual sensitivity (see Li et al., 2007) to expect feedback to be more negative. From another angle, increased P1 amplitudes in anticipation of both negative and more relevant social evaluative feedback are known (Schindler et al., 2019). Concerning the positive relationship between the FRN and positive expectations, this seems compatible with the reward-related interpretation of this time window and sensor cluster (Proudfit, 2015). However, please note that these correlations are exploratory (twenty-five calculated correlations), but future studies may use these findings as predictors of selective ERP-behavior relationships.

## Full list of the Individual Random Effects Coefficients

We added the point estimate (*b*) for each individual participant with the variables coded for sender relevance (Relevance), sender valence (Valence), and trial repetition number (RepetitionNo).

|  | (Intercept) | Relevance | Valence | RepetitionNo | Relevance:Valence | Relevance:RepetitionNo | Valence:RepetitionNo | Relevance:Valence:RepetitionNo |
| --- | --- | --- | --- | --- | --- | --- | --- | --- |
|  | ***b*** | | | | | | | |
| HC000 | .739 | -.138 | -.824 | -.011 | .428 | -.006 | -.033 | -.026 |
| HC001 | -.317 | -.130 | -.235 | .009 | -.617 | .017 | .013 | .014 |
| HC002 | -.014 | -.691 | -.194 | -.003 | .005 | .010 | -.002 | .008 |
| HC003 | -.062 | .385 | .508 | .017 | .031 | -.002 | .000 | -.024 |
| HC004 | -.264 | -.024 | -.258 | -.010 | -.268 | -.005 | .007 | .019 |
| HC005 | .034 | -.094 | -.355 | -.003 | -.203 | .004 | .001 | .007 |
| HC006 | .206 | .591 | .072 | .007 | -.321 | -.004 | .005 | .011 |
| HC007 | -.578 | -.284 | .210 | .009 | -.222 | .011 | .011 | -.006 |
| HC008 | -.151 | -.396 | .033 | .006 | -.283 | .011 | .007 | .015 |
| HC009 | .017 | .256 | .021 | .021 | -.495 | .007 | -.001 | -.010 |
| HC010 | -.527 | .292 | .519 | .013 | -.157 | .000 | .012 | -.014 |
| HC011 | .058 | -.114 | -.017 | -.004 | .054 | -.001 | .000 | .006 |
| HC012 | .484 | .740 | -1.004 | .002 | -1.258 | .000 | .002 | .059 |
| HC013 | .173 | .095 | -.099 | -.006 | .227 | -.005 | -.006 | -.005 |
| HC014 | .111 | .281 | -.313 | .001 | -.588 | .003 | .008 | .034 |
| HC015 | -.275 | .076 | .910 | .015 | .873 | -.003 | -.011 | -.069 |
| HC016 | .344 | -.512 | -.117 | -.018 | .242 | .005 | .006 | .038 |
| HC017 | .366 | .198 | .005 | -.002 | -.516 | .000 | .015 | .056 |
| HC018 | -.103 | .876 | .399 | .002 | .084 | -.015 | .004 | -.007 |
| HC019 | -.039 | -.207 | -.131 | .004 | -.100 | .003 | -.004 | -.012 |
| HC020 | .098 | -.293 | .077 | -.005 | .186 | .009 | .005 | .017 |
| HC021 | .235 | -.149 | .068 | -.011 | -.003 | -.002 | .009 | .039 |
| HC023 | -.060 | .257 | .260 | .004 | .617 | -.007 | -.016 | -.051 |
| HC024 | -.223 | -.361 | .544 | -.002 | .288 | .005 | .012 | .008 |
| HC025 | .201 | -.036 | .056 | -.001 | .193 | .002 | -.002 | .002 |
| HC027 | .608 | -.081 | -.025 | -.011 | -.030 | .000 | .007 | .049 |
| HC029 | .246 | -.150 | .211 | -.008 | 1.135 | -.015 | -.027 | -.061 |
| HC030 | .105 | -.041 | .267 | -.009 | .902 | -.013 | -.016 | -.040 |
| HC032 | -.221 | -1.303 | -.161 | .007 | .633 | .021 | -.020 | -.066 |
| HC033 | -.814 | .125 | .270 | -.002 | .494 | -.008 | -.004 | -.050 |
| HC036 | -.212 | -.906 | -.066 | .003 | .520 | .009 | -.018 | -.053 |
| HC037 | .140 | .694 | .267 | .000 | .146 | -.016 | -.003 | -.004 |
| HC038 | -.505 | -.076 | -.021 | .007 | -.981 | .013 | .028 | .048 |
| HC040 | -.021 | -.136 | -.991 | -.012 | -.283 | -.001 | -.012 | .002 |
| HC041 | -.122 | -.196 | -.168 | .009 | -.512 | .004 | .001 | .005 |
| HC042 | -.128 | .093 | -.036 | -.007 | -.101 | .004 | .013 | .023 |
| HC044 | .201 | .338 | .241 | -.009 | .253 | -.010 | .004 | .015 |
| HC045 | .324 | .679 | -.182 | .002 | -.031 | -.014 | -.013 | -.011 |
| HC046 | .116 | .092 | .209 | .000 | -.151 | -.001 | .009 | .023 |
| HC048 | -.171 | .252 | .050 | -.003 | -.191 | -.009 | .004 | .015 |

1. **Full list of German original and translated adjectives**

| List | Original adjective | Translation | Valence | List | Original adjective | Translation | | Valence |
| --- | --- | --- | --- | --- | --- | --- | --- | --- |
| 1 | ausgeglichen | balanced | 7,52 | 2 | ausdauernd | persistent | | 6,67 |
| 1 | ausgelassen | exuberant | 6,20 | 2 | überheblich | arrogant | | 2,46 |
| 1 | entspannt | relaxed | 7,23 | 2 | nachsichtig | indulgent | | 6,57 |
| 1 | bestimmend | determined | 3,56 | 2 | unbeweglich | immobile | | 3,45 |
| 1 | nachlässig | careless | 2,69 | 2 | weitsichtig | far-sighted | | 7,38 |
| 1 | überlegt | thoughtful | 6,82 | 2 | professionell | professional | | 7,66 |
| 1 | zerstörerisch | destructive | 1,93 | 2 | geradlinig | straightforward | | 5,88 |
| 1 | konsequent | consistent | 6,65 | 2 | intolerant | intolerant | | 2,07 |
| 1 | resigniert | resigned | 3,37 | 2 | paranoid | paranoid | | 2,43 |
| 1 | unzugänglich | inaccessible | 2,83 | 2 | anständig | decent | | 6,67 |
| 1 | manipulativ | manipulative | 2,04 | 2 | vielseitig | versatile | | 7,60 |
| 1 | feindselig | hostile | 2,00 | 2 | kommunikativ | communicative | | 7,39 |
| 1 | seriös | serious | 6,22 | 2 | respektlos | disrespectful | | 1,68 |
| 1 | willkürlich | arbitrary | 3,19 | 2 | intrigant | intriguing | | 2,11 |
| 1 | charismatisch | charismatic | 7,12 | 2 | diszipliniert | disciplined | | 7,18 |
| 1 | unangenehm | unpleasant | 2,56 | 2 | kompliziert | complicated | | 3,28 |
| 1 | eigenmächtig | high-handed | 4,56 | 2 | rücksichtslos | ruthless | | 1,79 |
| 1 | begehrenswert | desirable | 6,46 | 2 | selbstsüchtig | selfish | | 1,93 |
| 1 | redegewandt | eloquent | 6,93 | 2 | reizend | charming | | 6,47 |
| 1 | sorglos | carefree | 5,00 | 2 | stilvoll | stylish | | 7,00 |
| 1 | facettenreich | multifaceted | 6,85 | 2 | langweilig | boring | | 2,96 |
| 1 | sportlich | athletic | 7,93 | 2 | geduldig | patient | | 7,55 |
| 1 | achtsam | attentive | 6,93 | 2 | innovativ | innovative | | 7,47 |
| 1 | motivierend | motivating | 7,80 | 2 | großzügig | generous | | 7,27 |
| 1 | mutlos | despondent disheartened | 3,00 | 2 | sparsam | thrifty | | 5,26 |
| 1 | solidarisch | solidary | 7,55 | 2 | irrational | irrational | | 3,30 |
| 1 | arrogant | arrogant | 2,19 | 2 | erfinderisch | inventive | | 6,94 |
| 1 | unhöflich | rude | 2,31 | 2 | weltoffen | cosmopolitan | | 7,97 |
| 1 | ziellos | aimless | 2,96 | 2 | organisiert | organized | | 7,25 |
| 1 | realistisch | realistic | 6,73 | 2 | faszinierend | fascinating | | 7,29 |
| 1 | zuverlässig | reliable | 8,34 | 2 | fleißig | hardworking | | 7,61 |
| 1 | argwöhnisch | suspicious | 3,14 | 2 | wehleidig | snivelling | | 2,61 |
| 1 | ambitioniert | ambitious | 7,25 | 2 | dominant | dominant | | 3,83 |
| 1 | unnahbar | unapproachable | 3,48 | 2 | idealistisch | idealistic | | 5,31 |
| 1 | dynamisch | dynamic | 6,53 | 2 | originell | original | | 6,56 |
| 1 | talentiert | talented | 7,69 | 2 | tolerant | tolerant | | 7,62 |
| 1 | wütend | angry | 2,61 | 2 | tapfer | brave | | 7,03 |
| 1 | witzig | funny | 7,41 | 2 | hilfsbereit | helpful | | 7,79 |
| 1 | skrupellos | ruthless | 1,87 | 2 | hoffnungslos | hopeless | | 2,38 |
| 1 | bodenständig | down-to-earth | 6,90 | 2 | boshaft | malicious | | 1,67 |
| 1 | wagemutig | daring | 6,18 | 2 | fantasievoll | imaginative | | 6,48 |
| 1 | lernwillig | willing to learn | 7,37 | 2 | kooperativ | cooperative | | 7,83 |
| 1 | optimistisch | optimistic | 7,90 | 2 | dekadent | decadent | | 4,19 |
| 1 | pünktlich | punctual | 7,25 | 2 | egoistisch | | selfish | 2,19 |
| 1 | lügnerisch | lying | 1,60 | 2 | ekelerregend | disgusting | | 1,67 |
| 3 | hinterhältig | sneaky | 1,81 | 4 | schlagfertig | quick-headed | | 6,89 |
| 3 | entschlossen | determined | 6,67 | 4 | schadenfroh | gloating | | 2,77 |
| 3 | nachtragend | resentful | 2,50 | 4 | überzeugend | convincing | | 6,57 |
| 3 | ungeduldig | impatient | 3,32 | 4 | angeberisch | boastful | | 2,58 |
| 3 | standhaft | steadfast | 6,64 | 4 | geschickt | skillful | | 7,15 |
| 3 | distanziert | distant | 3,31 | 4 | unterhaltsam | entertaining | | 7,10 |
| 3 | strukturiert | structured | 6,97 | 4 | strebsam | ambitious | | 6,65 |
| 3 | berechenbar | predictable | 3,93 | 4 | intelligent | intelligent | | 7,96 |
| 3 | umsichtig | prudent | 6,78 | 4 | belastbar | resilient | | 7,07 |
| 3 | neurotisch | neurotic | 3,00 | 4 | gewissenhaft | conscientious | | 7,81 |
| 3 | selbstbewusst | self-confident | 7,58 | 4 | pragmatisch | pragmatic | | 6,17 |
| 3 | bedrohlich | threatening | 2,19 | 4 | behutsam | cautious | | 6,86 |
| 3 | abweisend | dismissive | 2,47 | 4 | selbstgerecht | self-righteous | | 3,48 |
| 3 | launisch | moody | 2,55 | 4 | beliebt | popular | | 6,63 |
| 3 | gefallsüchtig | eager to | 2,79 | 4 | individuell | individual | | 6,86 |
| 3 | kritikfähig | criticize | 7,25 | 4 | charmant | charming | | 7,34 |
| 3 | musikalisch | musical | 6,67 | 4 | chaotisch | chaotic | | 3,57 |
| 3 | sprachbegabt | linguistic gifted | 7,50 | 4 | großkotzig | big-headed | | 1,57 |
| 3 | oberflächlich | superficial | 2,55 | 4 | harmonisch | harmonious | | 7,23 |
| 3 | kompetent | competent | 7,97 | 4 | authentisch | authentic | | 7,73 |
| 3 | modisch | fashionable | 5,86 | 4 | willensstark | strong-willed | | 7,07 |
| 3 | bezaubernd | charming | 7,87 | 4 | selbstsicher | self-confident small | | 7,07 |
| 3 | sanftmütig | gentle | 6,93 | 4 | kleinkariert | small-minded | | 3,11 |
| 3 | lebensfroh | cheerful | 8,30 | 4 | feinfühlig | sensitive | | 6,96 |
| 3 | liebevoll | loving | 8,14 | 4 | dickköpfig | stubborn | | 3,11 |
| 3 | souverän | confident | 7,30 | 4 | reflektierend | reflective | | 8,25 |
| 3 | eigenständig | independent | 7,36 | 4 | flexibel | flexible | | 7,27 |
| 3 | kreativ | creative | 7,67 | 4 | brillant | brilliant | | 7,42 |
| 3 | sensibel | sensitive | 5,33 | 4 | elegant | elegant | | 6,90 |
| 3 | humorvoll | humorous | 8,04 | 4 | anmaßend | arrogant | | 2,67 |
| 3 | wertvoll | valuable | 7,67 | 4 | zielstrebig | determined | | 7,53 |
| 3 | wahnsinnig | mad | 3,25 | 4 | engagiert | committed | | 7,90 |
| 3 | sympathisch | sympathetic | 7,90 | 4 | deprimiert | depressed | | 2,33 |
| 3 | fürsorglich | caring | 7,72 | 4 | garstig | nasty | | 2,57 |
| 3 | spontan | spontaneous | 6,92 | 4 | wachsam | watchful | | 6,07 |
| 3 | negativ | negative | 2,22 | 4 | frustriert | frustrated | | 2,70 |
| 3 | diplomatisch | diplomatic | 6,93 | 4 | warmherzig | warm-hearted | | 8,04 |
| 3 | makellos | flawless | 5,48 | 4 | habgierig | greedy | | 1,89 |
| 3 | aggressiv | aggressive | 1,61 | 4 | fröhlich | cheerful | | 8,04 |
| 3 | jähzornig | irascible | 1,83 | 4 | cholerisch | choleric | | 1,97 |
| 3 | egozentrisch | self-centered | 2,39 | 4 | ignorant | ignorant | | 2,00 |
| 3 | teamfähig | team player | 7,78 | 4 | elitär | elitist | | 3,79 |
| 3 | zögerlich | hesitant | 3,97 | 4 | waghalsig | reckless | | 4,46 |
| 3 | hysterisch | hysterical | 1,93 | 4 | eifersüchtig | | jealous | 2,47 |

1. **Supplementary References**

Abdel Rahman, R. (2011). Facing good and evil: Early brain signatures of affective biographical knowledge in face recognition. *Emotion (Washington, D.C.)*, *11*(6), 1397–1405. https://doi.org/10.1037/a0024717

Baum, J., & Abdel Rahman, R. (2021). Negative news dominates fast and slow brain responses and social judgments even after source credibility evaluation. *NeuroImage*, *244*, 118572. https://doi.org/10.1016/j.neuroimage.2021.118572

Baum, J., Rabovsky, M., Rose, S. B., & Abdel Rahman, R. (2018). Clear judgments based on unclear evidence: Person evaluation is strongly influenced by untrustworthy gossip. *Emotion (Washington, D.C.)*. https://doi.org/10.1037/emo0000545

Brown, C., El-Deredy, W., & Blanchette, I. (2010). Attentional modulation of visual-evoked potentials by threat: Investigating the effect of evolutionary relevance. *Brain and Cognition*, *74*(3), 281–287. https://doi.org/10.1016/j.bandc.2010.08.008

Dolcos, F., & Cabeza, R. (2002). Event-related potentials of emotional memory: Encoding pleasant, unpleasant, and neutral pictures. *Cognitive, Affective & Behavioral Neuroscience*, *2*, 252-63.

Giménez-Fernández, T., Kessel, D., Fernández-Folgueiras, U., Fondevila, S., Méndez-Bértolo, C., Aceves, N., García-Rubio, M. J., & Carretié, L. (2020). Prejudice drives exogenous attention to outgroups. *Social Cognitive and Affective Neuroscience*, *15*(6), 615–624. https://doi.org/10.1093/scan/nsaa087

Gupta, R. S., Kujawa, A., & Vago, D. R. (2019). The neural chronometry of threat-related attentional bias: Event-related potential (ERP) evidence for early and late stages of selective attentional processing. *International Journal of Psychophysiology*, *146*, 20–42. https://doi.org/10.1016/j.ijpsycho.2019.08.006

Hajcak, G., MacNamara, A., & Olvet, D. M. (2010). Event-Related Potentials, Emotion, and Emotion Regulation: An Integrative Review. *Developmental Neuropsychology*, *35*(2), 129–155. https://doi.org/10.1080/87565640903526504

Kissler, J., & Strehlow, J. (2017). Something always sticks? How emotional language modulates neural processes involved in face encoding and recognition memory. *Poznan Studies in Contemporary Linguistics*, *53*(1), 63–93. https://doi.org/10.1515/psicl-2017-0004

Klimesch, W., Sauseng, P., & Hanslmayr, S. (2007). EEG alpha oscillations: The inhibition–timing hypothesis. *Brain Research Reviews*, *53*(1), 63–88.

Krasowski, C., Schindler, S., Bruchmann, M., Moeck, R., & Straube, T. (2021). Electrophysiological responses to negative evaluative person-knowledge: Effects of individual differences. *Cognitive, Affective & Behavioral Neuroscience*, *21*(4), 822–836. https://doi.org/10.3758/s13415-021-00894-w

Kroczek, L. O. H., & Mühlberger, A. (2022). Returning a smile: Initiating a social interaction with a facial emotional expression influences the evaluation of the expression received in return. *Biological Psychology*, *175*, 108453. https://doi.org/10.1016/j.biopsycho.2022.108453

Li, W., Zinbarg, R. E., & Paller, K. A. (2007). Trait anxiety modulates supraliminal and subliminal threat: Brain potential evidence for early and late processing influences. *Cognitive, Affective, & Behavioral Neuroscience*, *7*(1), 25–36. https://doi.org/10.3758/CABN.7.1.25

Luo, Q. L., Wang, H. L., Dzhelyova, M., Huang, P., & Mo, L. (2016). Effect of Affective Personality Information on Face Processing: Evidence from ERPs. *Frontiers in Psychology*, *7*. https://doi.org/10.3389/fpsyg.2016.00810

Proudfit, G. H. (2015). The reward positivity: From basic research on reward to a biomarker for depression. *Psychophysiology*, *52*(4), 449–459. https://doi.org/10.1111/psyp.12370

Qi, Y., Gu, R., Cao, J., Bi, X., Wu, H., & Liu, X. (2017). Response bias-related impairment of early subjective face discrimination in social anxiety disorders: An event-related potential study. *Journal of Anxiety Disorders*, *47*, 10–20. https://doi.org/10.1016/j.janxdis.2017.02.003

Schindler, S., Bruchmann, M., Krasowski, C., Moeck, R., & Straube, T. (2021). Charged With a Crime: The Neuronal Signature of Processing Negatively Evaluated Faces Under Different Attentional Conditions. *Psychological Science*, *32*(8), 1311–1324. https://doi.org/10.1177/0956797621996667

Schindler, S., Bruchmann, M., & Straube, T. (2023). Beyond facial expressions: A systematic review on effects of emotional relevance of faces on the N170. *Neuroscience & Biobehavioral Reviews*, *153*, 1–23. https://doi.org/10.1016/j.neubiorev.2023.105399

Schindler, S., Vormbrock, R., & Kissler, J. (2019). Emotion in Context: How Sender Predictability and Identity Affect Processing of Words as Imminent Personality Feedback. *Frontiers in Psychology*, *10*, 94. https://doi.org/10.3389/fpsyg.2019.00094

Slagter, H. A., Prinssen, S., Reteig, L. C., & Mazaheri, A. (2016). Facilitation and inhibition in attention: Functional dissociation of pre-stimulus alpha activity, P1, and N1 components. *NeuroImage*, *125*, 25–35. https://doi.org/10.1016/j.neuroimage.2015.09.058

Suess, F., Rabovsky, M., & Abdel Rahman, R. (2015). Perceiving emotions in neutral faces: Expression processing is biased by affective person knowledge. *Social Cognitive and Affective Neuroscience*, *10*(4), 531–536. https://doi.org/10.1093/scan/nsu088

Xu, M., Li, Z., Diao, L., Fan, L., & Yang, D. (2016). Contextual Valence and Sociality Jointly Influence the Early and Later Stages of Neutral Face Processing. *Frontiers in Psychology*, *7*, 1258. https://doi.org/10.3389/fpsyg.2016.01258
